# First report of monogenean flatworms from Lake Tana, Ethiopia: gill parasites of the commercially important *Clarias gariepinus* (Teleostei: Clariidae) and *Oreochromis niloticus tana* (Teleostei: Cichlidae)

**DOI:** 10.1186/s13071-016-1691-2

**Published:** 2016-07-25

**Authors:** Moges Beletew, Abebe Getahun, Maarten P. M. Vanhove

**Affiliations:** 1 Department of Biology, College of Natural and Computational Sciences, Debre Markos University, P.O.Box: 269, Debre Markos, Ethiopia; 2 Fisheries and Aquatic Sciences Stream, Department of Zoological Sciences, Addis Ababa University, P.O.Box: 1176, Addis Ababa, Ethiopia; 3 Biology Department, Royal Museum for Central Africa, Leuvensesteenweg 13, 3080 Tervuren, Belgium; 4 Department of Botany and Zoology, Faculty of Science, Masaryk University, Kotlářská 2, 611 37 Brno, Czech Republic; 5 Laboratory of Biodiversity and Evolutionary Genomics, Department of Biology, University of Leuven, Charles Debériotstraat 32, 3000 Leuven, Belgium; 6 Institute of Marine Biological Resources and Inland Waters, Hellenic Centre for Marine Research, 46.7 km Athens-Sounio Avenue, PO Box 712, Anavyssos, 190 13 Greece; 7 Present address: Capacities for Biodiversity and Sustainable Development, Operational Directorate Natural Environment, Royal Belgian Institute of Natural Sciences, Vautierstraat 29, 1000 Brussels, Belgium

**Keywords:** *Cichlidogyrus*, Dactylogyridea, Gyrodactylidea, *Gyrodactylus*, *Macrogyrodactylus*, Monogenea, Perciformes, *Scutogyrus*, Siluriformes, *Quadriacanthus*

## Abstract

**Background:**

Lake Tana is the largest lake in Ethiopia and the source of the Blue Nile. The lake harbours unique endemic cyprinid fish species, as well as the commercially important endemic Nile tilapia subspecies *Oreochromis niloticus tana* and the North African catfish *Clarias gariepinus*. Its endemicity, especially within the *Labeobarbus* radiation, its conservation importance and its economic indispensability attract scientific interest to the lake’s ichthyofauna. Fish parasites of Lake Tana, however, are hitherto poorly known, and no formal report exists on its monogenean flatworms. For sustainable aquaculture and fisheries development, it is essential to study monogenean fish parasites in these economically most important fish species. Moreover, it remains to be verified whether this unique ecosystem and its endemicity gave rise to a distinct parasite fauna as well.

**Results:**

Nile tilapia and North African catfish hosts were collected from Lake Tana in 2013. Nine species of monogenean parasites of two orders, Gyrodactylidea Bychowsky, 1937 and Dactylogyridea Bychowsky, 1937, were recovered. *Gyrodactylus gelnari* Přikrylová, Blažek & Vanhove, 2012, *Macrogyrodactylus clarii* Gussev, 1961, *Quadriacanthus aegypticus* El-Naggar & Serag*,* 1986 and two undescribed *Quadriacanthus* species were recovered from *C. gariepinus. Oreochromis niloticus tana* hosted *Cichlidogyrus cirratus* Paperna, 1964, *C. halli* (Price & Kirk, 1967), *C. thurstonae* Ergens, 1981 and *Scutogyrus longicornis* (Paperna & Thurston, 1969).

**Conclusions:**

Except for *M. clarii*, all species represent new records for Ethiopia. This first study on the monogenean fauna of Lake Tana revealed that the lake’s North African catfish, as well as its endemic Nile tilapia subspecies, harbour parasites that are known from these host species elsewhere in Africa.

## Background

**Fig. 1:**
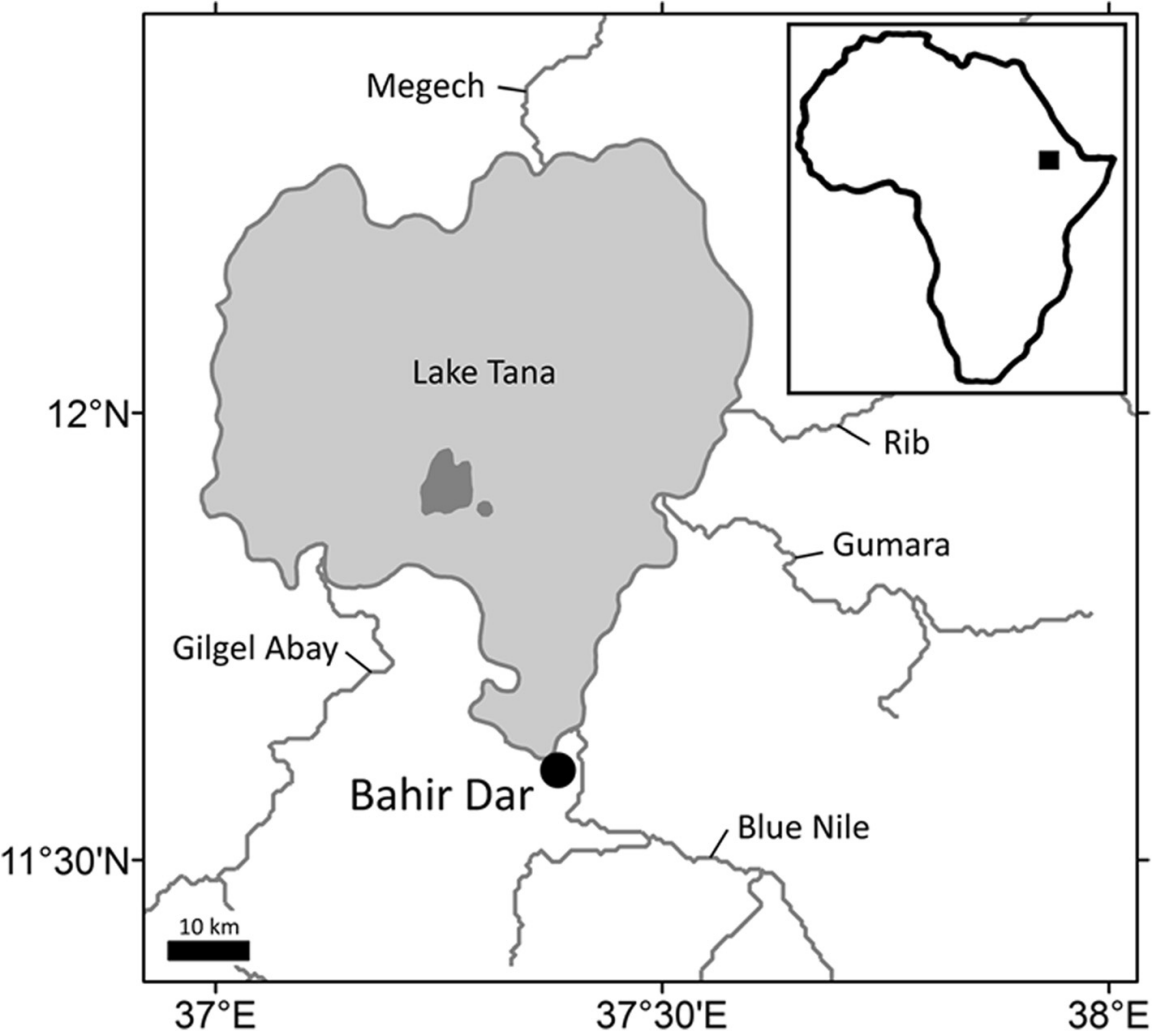
Lake Tana and its major tributaries. The study site (Bahir Dar) is indicated

Lake Tana is the largest lake in Ethiopia with a surface area of 3050 km^2^ and a maximum width and length of 68 and 78 km, respectively. It contains half the country’s freshwater resources and is the third largest lake in the Nile Basin. It is located at an altitude of 1830 m and is the source of the River Blue Nile. The lake was formed as a result of volcanic activity blocking the course of a number of rivers in the early Pleistocene, an estimated 5 million years ago. It is fed by perennial and intermittent rivers. Of the major perennial rivers (Megech, Gumara, Rib and Gilgel Abay) that feed the lake, Gilgel Abay (Little Blue Nile) is the largest one. These rivers supply more than 95 % of the total annual inflow to the lake (see [1] and references therein) (Fig. 1).

Generally four fish families are native to the lake: Nemacheilidae, Cichlidae, Clariidae and Cyprinidae. Nemacheilidae, Clariidae and Cichlidae are represented only by one species each, respectively *Afronemacheilus abyssinicus* (Boulenger, 1902), the North African catfish *Clarias gariepinus* (Burchell, 1822) and *Oreochromis niloticus tana* Seyoum & Kornfield, 1992, a subspecies of the Nile tilapia *Oreochromis niloticus* (Linnaeus, 1758). Of the 28 fish species in Lake Tana, 21 are endemic. Of these endemics, 19 are cyprinids and two are non-cyprinids: *Afronemacheilus abyssinicus* (Boulenger, 1902) and *Oreochromis niloticus tana* [2]. The largest fish family in the lake is the Cyprinidae, represented by four genera, i.e. *Varicorhinus* Rüppel, 1835 (one species: *Varicorhinus beso* Rüppel, 1835), *Labeobarbus* Rüppell, 1835 (16 species), *Barbus* Cuvier & Cloquet, 1816 (three species) and *Garra* Hamilton, 1822 (four species) [2, 3]. The 16 *Labeobarbus* morphotypes are the World’s only species flock of large cyprinids, after anthropogenic near-annihilation of the one within Lake Lanao (Philippines) [4, 5]. Thus, Lake Tana is considered a living evolutionary laboratory [1] and by virtue of its unique fish and bird biodiversity, the lake has been recognised as one of the global top 250 lakes in terms of conservation priority [6].

As compared to the numerous ichthyological studies carried out in the lake and its tributaries (e.g. [3–5, 7]), parasitological work in the lake is scarce. While detailed accounts of digeneans and cestodes infecting Lake Tana’s fishes exist, these did not focus on the unique evolutionary position of the lake’s biodiversity [8–15]. Particularly, monogeneans are scientifically completely untouched in formal reports. These mostly ectoparasitic flatworms, by virtue of their species richness, simple one-host life-cycles, and high host-specificity, hence with a close relationship to their host species, are interesting models for evolutionary research into host-parasite relations. Assemblages of closely related fishes, like in cichlids or other species flocks, provide an ideal setting to study monogenean diversity and evolution [16]. Work on the Monogenea from Africa began many decades ago, with the discovery of *Macrogyrodactylus* Malmberg, 1957 [17] and since then numerous monogenean parasites have been recorded. With regard to parasitological studies, and even more so when specifically referring to monogeneans, Africa is still insufficiently explored and new genera are regularly reported from African freshwater fishes, e.g. [18, 19]. Relatively widely studied host fish taxa include catfishes (e.g. [20–23] for overviews) and cichlids (e.g. [24]).

African clariids harbour monogenean species in the dactylogyridean genera *Quadriacanthus* Paperna, 1961 and *Paraquadriacanthus* Ergens, 1988 [20, 21] and in the gyrodactylidean genera *Gyrodactylus* von Nordmann, 1832 and *Macrogyrodactylus* [22, 23]*.* Of these, only *Macrogyrodactylus clarii* Gussev, 1961 was recorded in Ethiopia. Of the cichlid monogenean parasites found in Africa so far, the dactylogyridean *Cichlidogyrus* Paperna, 1960 is the most dominant and diverse genus [24] and the number of formally described species has increased to about 100 ([25] and references therein). *Cichlidogyrus* species richness on the host and their specificity exhibit considerable variation [24]. Within Gyrodactylidea, species of *Gyrodactylus* have been described from African cichlids throughout the continent, including in Ethiopia, where *G. hildae* García-Vásquez, Hansen, Christison, Bron & Shinn, 2011 was described from *O. niloticus niloticus* [26].

North African catfish and (Nile) tilapia are economically important species and they are the most common species in the aquaculture industry in sub-Saharan Africa [27]. They have been spread worldwide primarily for aquaculture. This study is conducted in view of the potential impact of monogenean parasites on catfish and tilapia culture [28]. Identifying and inventorying the monogeneans of two of Lake Tana’s most commercially important fish species is relevant to aquaculture and fisheries development in the lake region as well as in Ethiopia as a whole. These are considered as high-potential but underexploited sectors to meet protein demand [29]. In view of the high proportion of endemism in Lake Tana’s ichthyofauna, we will also test whether the unique Lake Tana environment gave rise to a unique gill monogenean fauna, or whether the two target species are infected by the same parasites in Lake Tana as elsewhere in Africa.

## Methods

Nile Tilapia and North African catfish monogeneans were collected during October-November 2013. The live hosts were obtained from fishermen at the Corporation Area in the city of Bahir Dar (Fig. 1). This is the main landing site, where fish is also filleted for market use and household consumption. In the laboratory, gills were removed and inspected in a Petri dish with lake water. Parasites were isolated using parasitological needles and brushes following standard procedures and fixed under a coverslip using glycerine ammonium picrate. Identification of monogeneans, based on the hard parts of the attachment organ (haptor), vagina and male copulatory organ (MCO) and according to [20, 22–24], was carried out with an Olympus BX51 phase contrast microscope. Parasite voucher specimens were deposited in the invertebrate collection of the Royal Museum for Central Africa (RMCA), Tervuren, Belgium (see accession numbers in Table 1).

**Table 1:** The monogenean parasite species recovered from *Clarias gariepinus* and *Oreochromis niloticus tana* in Lake Tana

Parasite order	Parasite genus	Parasite species	Host species	RMCA voucher
Gyrodactylidea Bychowsky, 1937	*Gyrodactylus* von Nordmann, 1832	*Gyrodactylus gelnari* Přikrylová, Blažek & Vanhove, 2012	*Clarias gariepinus* (Burchell, 1822)	MRAC MT 37808; MRAC MT 37824
	*Macrogyrodactylus* Malmberg, 1957	*Macrogyrodactylus clarii* Gussev, 1961	*C. gariepinus*	MRAC MT 37807; MRAC MT 37823
Dactylogyridea Bychowsky, 1937	*Cichlidogyru*s Paperna, 1960	*Cichlidogyrus cirratus* Paperna, 1964	*Oreochromis niloticus tana* Seyoum & Kornfield, 1992	MRAC MT 37805; MRAC MT 37820-21
		*Cichlidogyrus halli* (Price & Kirk, 1967)	*O. niloticus tana*	MRAC MT 37804; MRAC MT 37819
		*Cichlidogyrus thurstonae* Ergens, 1981	*O. niloticus tana*	MRAC MT 37803; MRAC MT 37818
	*Scutogyrus* Pariselle & Euzet, 1995	*Scutogyrus longicornis* (Paperna & Thurston, 1969)	*O. niloticus tana*	MRAC MT 37806; MRAC MT 37822
	*Quadriacanthus* Paperna, 1961	*Quadriacanthus aegypticus* El-Naggar & Serag, 1986	*C. gariepinus*	MRAC MT 37809; MRAC MT 37825
		*Quadriacanthus* sp. 1	*C. gariepinus*	MRAC MT 37810; MRAC MT 37826
		*Quadriacanthus* sp. 2	*C. gariepinus*	MRAC MT 37811

*Abbreviations*: *RMCA* Royal Museum for Central Africa, *MRAC MT* Musée royal de l’Afrique centrale – Musée de Tervuren

## Results

**Fig. 2:**
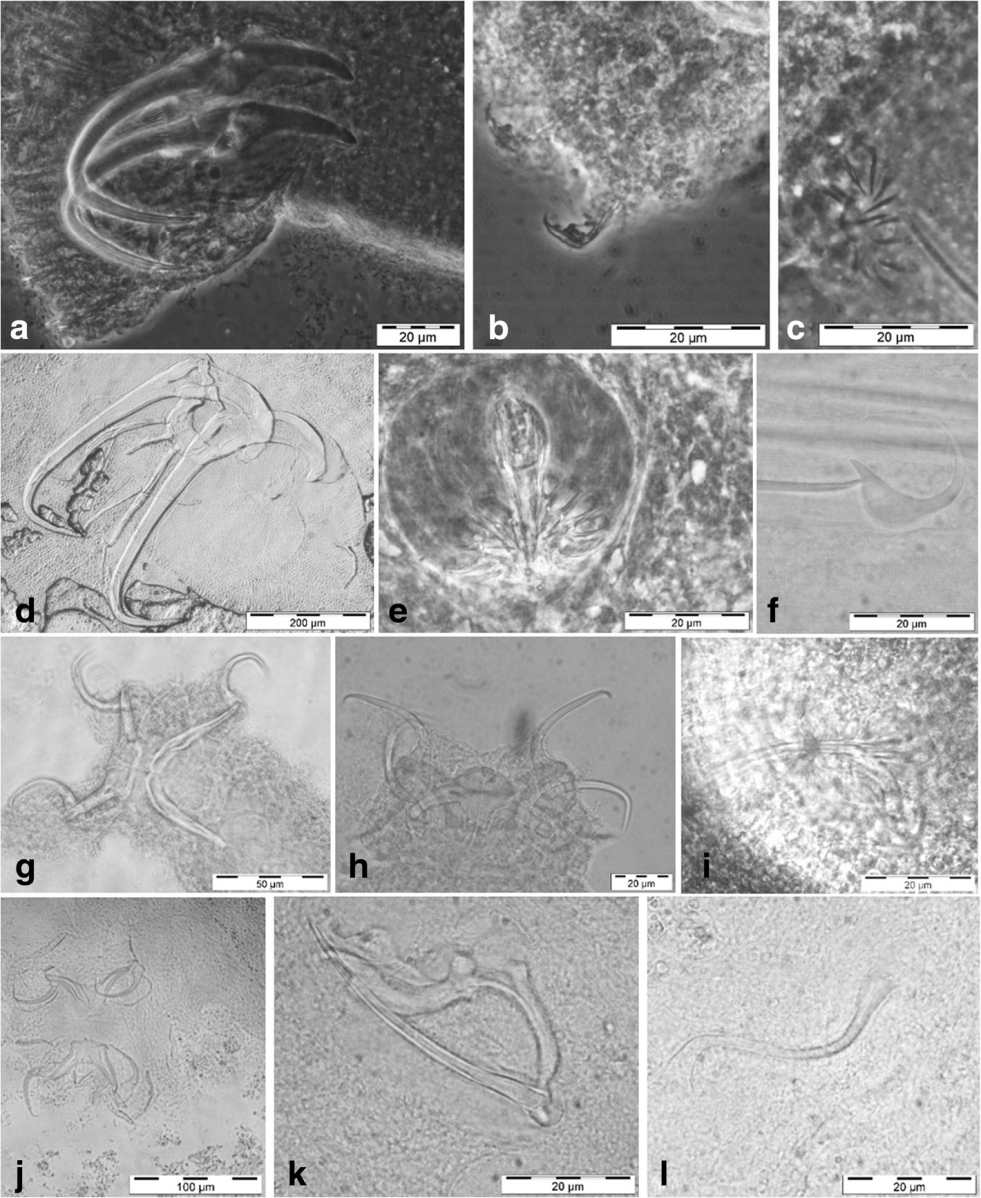
Photomicrographs of haptoral and genital hard parts of the monogenean species collected in Lake Tana from the North African catfish *Clarias gariepinus*. **a** *Gyrodactylus gelnari*, haptor. **b** *G. gelnari*, marginal hook sickles. **c** *G. gelnari*, spines of the male copulatory organ. **d** *Macrogyrodactylus clarii*, haptor. **e** *M. clarii*, spines of the male copulatory organ. **f** *M. clarii*, marginal hook sickle. **g** *Quadriacanthus* sp. 1, haptor. **h** *Quadriacanthus* sp. 2, haptor. **i** *Quadriacanthus* sp. 2, male copulatory organ. **j** *Quadriacanthus aegypticus*, haptor. **k** *Q. aegypticus*, male copulatory organ. **l** *Q. aegypticus*, vagina

**Fig. 3:**
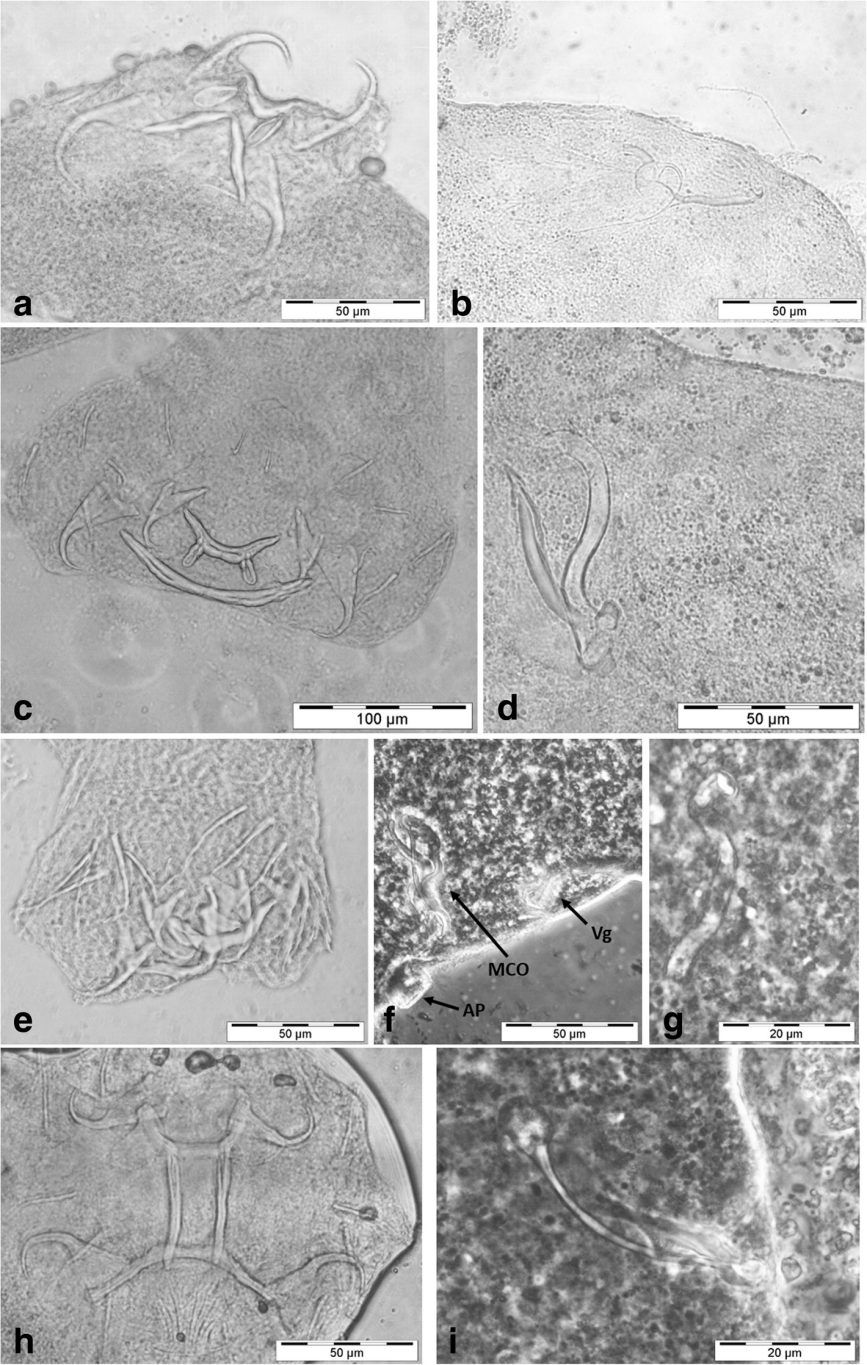
Photomicrographs of haptoral and genital hard parts of the monogenean species collected from the endemic subspecies of Nile tilapia, *Oreochromis niloticus tana*. **a** *Cichlidogyrus cirratus*, haptor. **b** *C. cirratus*, male copulatory organ. **c** *Cichlidogyrus halli*, haptor. **d** *C. halli*, male copulatory organ. **e** *Cichlidogyrus thurstonae*, haptor. **f** *C. thurstonae*, genital hard parts, arrows indicate male copulatory organ (MCO), vagina (Vg) and auxiliary plate (AP). **g** *Scutogyrus longicornis*, vagina. **h** *S. longicornis*, haptor. **i** *S. longicornis*, male copulatory organ

All five inspected Nile tilapia specimens were infected by 13–32 monogeneans, with a mean intensity of 19.3 monogeneans/fish. Five of seven catfish investigated harboured 5–7 monogeneans at a mean intensity of 6 monogeneans/fish. A total of nine gill monogenean species were found, representing both dactylogyrideans and gyrodactylideans (Table 1, Figs. 2 and 3). Specimens belonging to *Gyrodactylus*, *Macrogyrodactylus* and *Quadriacanthus*, totaling five species, were recovered from *Clarias gariepinus*. Representatives of the latter parasite genus included two undescribed species. The Nile tilapia harboured four species within two genera: *Cichlidogyrus* and *Scutogyrus* Pariselle & Euzet, 1995 (Table 1).

## Discussion

Lake Tana is one of the major fishing grounds in Ethiopia, supplying cheap protein to the local communities as well as to main cities such as Addis Ababa. Nile tilapia and North African catfish are the most vital and preferred species in the fisheries and aquaculture activities. This research was conducted to explore the monogenean parasites of the commercially valuable catfish and tilapia in Lake Tana, and to test whether this unique ecosystem harbours an equally distinctive parasite fauna. Nine monogenean species were found, all of which are new records for the lake; except for *M. clarii*, all are also new to the country’s biodiversity.

The genus *Macrogyrodactylus* currently includes nine species. In the present study, *Macrogyrodactylus clarii* was recorded from *Clarias gariepinus*. This species was described from the gills of *Clarias* sp. from an unspecified location in Ethiopia; hence this is the first record from Lake Tana. The parasite was also recovered from different clariid hosts throughout Africa [22, 30]. Catfishes are infected by over 20 monogenean species belonging to *Quadriacanthus*, some of which are host-specific while others are shared between hosts [21]. *Clarias gariepinus* in Lake Tana was infected with three *Quadriacanthus* species, including *Q. aegypticus* El-Naggar & Serag, 1986, widespread on the same host in Africa, and two undescribed species, here designated as *Quadriacanthus* sp. 1 and *Quadriacanthus* sp. 2. The haptor of the former (Fig. 2g) is somewhat reminiscent of *Quadriacanthus simplex* N’Douba, Lambert & Euzet, 1999 [31], in view of the anchor shapes: the ventral anchors with curved shaft and long point, the dorsal ones with a long point at an almost perpendicular angle. *Quadriacanthus* sp. 2 is similar to *Q. clariadis* Paperna, 1961 and *Q. longifilisi* N’Douba, Lambert & Euzet, 1999 in anchor configuration, but deviates from previously described species mainly in its long, slender and slightly curved copulatory tube, of almost constant diameter but slightly wider at its base (Fig. 2i). As indeed several studies indicate the presence of undescribed *Quadriacanthus* species even on their well-studied clariid hosts (L. Šafarčíková, personal communication), further detailed morphological and molecular studies are needed to fully inventorise this parasite fauna and clarify the exact taxonomical status of its representatives. Regarding the species of *Gyrodactylus* infecting *Clarias gariepinus*, representatives of this host in Lake Tana harboured *G. gelnari* Přikrylová, Blažek & Vanhove, 2012. This species was described from the fins of Senegalese *Clarias anguillaris* (Linnaeus, 1758) [23].

The most dominant and diverse monogeneans on African cichlids are representatives of *Cichlidogyrus*. Three species were found in Lake Tana’s Nile tilapia: *Cichlidogyrus cirratus* Paperna, 1964, *C. halli* (Price & Kirk, 1967) and *C. thurstonae* Ergens, 1981*.* Besides these, the same host harboured *Scutogyrus longicornis* (Paperna & Thurston, 1969). These four species are the first monogeneans reported from the Nile tilapia subspecies endemic to the lake. They infect a wide range of cichlid hosts throughout Africa; some of these species have been anthropogenically co-introduced with tilapia in Asia or the Neotropics [24, 32, 33]. In view of the high diversity of subspecies described from Nile tilapia, especially in Ethiopia and Kenya where four and three of them, respectively, are indigenous, the question has been raised whether these subspecies differ in their monogenean fauna [26].

## Conclusions

The present study shows that the endemic Nile tilapia in Lake Tana harbours common monogenean species widely distributed in Africa, on other Nile tilapia subspecies as well as on other cichlids. *Clarias gariepinus* in Lake Tana is infected by widespread monogenean parasites of African clariids, while also harbouring two undescribed species. More specimens of these species are needed to allow a more detailed characterisation and formal description. As a further perspective in Lake Tana fish parasitology, it is recommended to conduct a detailed survey of the endemic fish species, with a particular focus on the cyprinid fauna, to explore their monogenean species diversity, speciation, specificity, distribution and phylogeny.

## Abbreviations

MCO, male copulatory organ; RMCA, Royal Museum for Central Africa (Tervuren, Belgium)
